# Chemical Composition and Bioactivity of Laboratory-Fermented Bee Pollen in Comparison with Natural Bee Bread

**DOI:** 10.3390/biom13071025

**Published:** 2023-06-22

**Authors:** Michał Miłek, Mateusz Mołoń, Monika Kula-Maximenko, Ewelina Sidor, Grzegorz Zaguła, Małgorzata Dżugan

**Affiliations:** 1 Department of Chemistry and Food Toxicology, Institute of Food Technology and Nutrition, University of Rzeszow, Ćwiklińskiej 1a St., 35-601 Rzeszów, Poland; ewelina.sidor.dokt@gmail.com (E.S.); mdzugan@ur.edu.pl (M.D.); 2 Institute of Biology, University of Rzeszów, 35-601 Rzeszów, Poland; mmolon@ur.edu.pl; 3 The Franciszek Górski Institute of Plant Physiology, Polish Academy of Sciences, 30-239 Krakow, Poland; m.kula@ifr-pan.edu.pl; 4 Doctoral School, University of Rzeszów, Rejtana 16c, 35-959 Rzeszów, Poland; 5 Department of Bioenergetics and Food Analysis, Institute of Food Technology and Nutrition, University of Rzeszow, Ćwiklińskiej 2D, 35-601 Rzeszów, Poland; gzagula@ur.edu.pl

**Keywords:** bee pollen, fermentation, bee bread, yeast model, oxidative stress, chemical composition, Raman spectroscopy

## Abstract

Bee bread is a valuable product obtained from the hive on a relatively small scale, while bee pollen is more easily available. Therefore, an effective laboratory method of converting pollen into a bee bread substitute is desired. The aim of the research was to verify the influence of selected factors (temperature, ultrasound) on the quality of obtained product using *Lactobacillus rhamnosus* inoculum. The composition of the fermented pollen was analyzed using Inductively Coupled Plasma Optical Emission Spectroscopy (ICP-OES), Raman spectroscopy, and SDS-PAGE and compared to natural bee bread and the original pollen. In vitro biological activity was assessed as antioxidant activity using a yeast model (BY4741 and *sod1∆* strains). Fermentation of pollen occurred spontaneously and after inoculation, as demonstrated by lower pH and higher lactic acid content. Raman spectroscopy and ICP-OES confirmed changes in composition compared to the initial pollen. Compared to bee bread, the fermented pollen showed a higher content of polyphenols and comparable antioxidant activity; moreover, it accelerated yeast growth rate. In addition, a protective effect was observed for Cu/Zn-superoxide dismutase 1 (*sod1∆* yeast mutant exposed to hydrogen peroxide-induced oxidative stress). The higher fermentation temperature (25 °C) produces a more bee-bread-like product, while the use of ultrasound and starter culture seems to have no positive effect.

## 1. Introduction

Bee pollen is plant floral pollen collected by honey bees with the help of a third pair of legs with pollen baskets. When collecting pollen, honey bees use nectar and regurgitated honey to moisten it, while their salivary gland secretions, rich in enzymes such as amylase, invertase, and glucosidase, help agglutinate the pollen and pack it. Then, the pollen is transported by bees to the hive where it is deposited in the cells of the comb [1,2]. Regardless of its origin and collection by different species of bees, bee pollen contains pollen grains made up of an outer layer of pollenkitt followed by layers of exine and intine surrounding the interior of the pollen. Exine, made of sporopollenin, is the most difficult element of the pollen grain to break down during its processing [3]. 

From pollen with the addition of honey, bees produce bee bread, which is the result of a complex fermentation process. At the temperature inside the hive, pollen is fermented by lactic acid bacteria (mainly *Lactobacillus* sp. and *Fructobacillus* sp.). As a result of fermentation, lactic acid is produced with a concentration of at least 3%, which further helps to preserve the bee bread [4]. Apart from bacteria, pollen microflora also contains various species of yeasts and molds, which can also participate in fermentation processes as well as microbial deterioration processes [5,6]. Like bee pollen, bee bread contains the same main chemical components, i.e., proteins, carbohydrates, and lipid substances (saturated and unsaturated fatty acids). It also contains other nutrients, such as minerals, carotenoids, phytohormones, and vitamins. A characteristic feature that distinguishes bee bread from pollen is the content of lactic acid, which is present in large amounts in bee bread and responsible for the low pH of this product [4,5]. The chemical composition of bee bread varies depending on its botanical origin, climatic conditions, geographic location, type of soil, or the possible unhygienic behavior of beekeepers during its collection and storage [5,6]. Bee bread can be considered a dietary supplement due to its high nutrient content, with better bioavailability than bee pollen constituents. Bee bread is an important source of various compounds (mainly phenolic substances), which show antioxidant, anticancer, antibacterial, and neuroprotective activity [7,8].

The production process of bee bread, i.e., the natural fermentation of pollen in the hive (in the cells of the comb), takes at least two weeks [9]. The purpose of the fermentation of bee pollen in laboratory conditions is to obtain a product (“artificial bee bread”) that will be more digestible and bioavailable in nutrients for the human body. This is due to the weakening of the outer layer of the field grain. It is postulated that during fermentation, the walls of pollen grains made of exine are partially degraded, which allows easier access and release of components present inside the grain [3,10,11]. There are known attempts to produce artificial bee bread by fermentation initiated by introducing lactic acid bacteria (LAB) into pollen [10,12,13,14]. Pollen fermentation with selected microbiological starter cultures is a biotechnological solution to the problems associated with the poor digestibility of pollen by humans. Moreover, obtaining pollen is well-known beekeeping practice, whereas collecting the bee bread is still more problematic and not commonly used. Bee bread is obtained in beekeeping conditions manually, with the use of special devices; sometimes mills are used on a larger scale but in these cases, the entire honeycomb is destroyed. Some research has focused on the optimization of fermentation conditions, i.e., the duration of fermentation, temperature, and selection of appropriate strains [12]. The methods of producing artificial bee bread allow for its production on a larger scale. Such a product could be used either directly as a functional food or as a component of functional fermented milk products, e.g., beverages, with added value compared to unfermented bee pollen [15]. Thus, the aim of the study was to examine how selected factors impact the laboratory process of bee pollen fermentation and to compare the properties of obtained fermented pollen with natural bee bread.

## 2. Materials and Methods

### 2.1. Material

#### 2.1.1. Bee Pollen and Bee Bread

One sample of multifloral bee pollen (P) as a starting substrate for fermentation was purchased from a local apiary (Podkarpackie, Poland). Five samples of natural bee bread (BB1–BB5) as a comparative material were purchased in various apiaries in the Podkarpackie Province (Poland).

#### 2.1.2. Microorganisms 

Lyophilized bacteria *Lactobacillus rhamnosus* GG (ATCC 53103) were used as a starter culture for the fermentation of bee pollen.

The budding yeast *Saccharomyces cerevisiae* strains used in this study were the wild-type haploid strain BY4741 (MATa *his3 leu2 met15 ura3*) and the isogenic mutant strain *sod1∆* (MATa *his3 leu2 met15 ura3 YJR104C:kanMX4*) (EUROSCARF, Oberursel, Germany).

### 2.2. Bee Pollen Treatment

Bee pollen (pollen loads) was ground into a fine powder using an MK-06M, Poland grinder (MPM, Milanówek, Poland). One batch of the samples was prepared from pollen treated with ultrasound (2 × 15 min, 700 W) using an ultrasonic bath (Sonic-10, Polsonic, Warsaw, Poland) in order to examine the influence of ultrasounds on the pollen structure and to demonstrate their possible sterilizing effect. 

### 2.3. Fermentation Process

**Figure 1:**
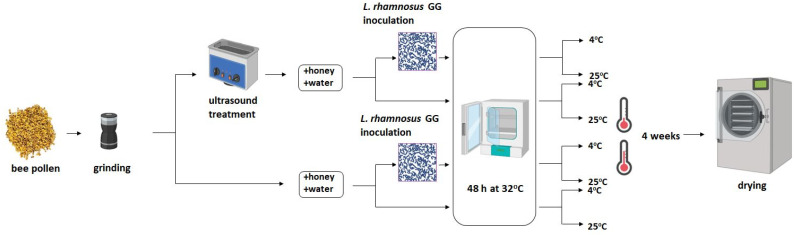
Experimental layout with applied variants of bee pollen treatment during fermentation.

Pollen fermentation was carried out according to Dany [16] with a slight modification. Fifty grams of ground bee pollen powder was mixed with 7.5 g of multiflorous honey and 12.5 mL of deionized water, and 1 g (3 × 10^9^ CFU) of freeze-dried bacterial culture was added in the case of inoculated samples. As control samples, mixtures of identical compositions were prepared but without inoculation with a culture of lactic acid bacteria (spontaneous fermentation). In addition, a series of ultrasound-treated pollen (see Section 2.2) samples were made in an identical arrangement. The mixtures were placed in sealed glass vessels (up to 2/3 of the volume) and initially placed in an incubator (32 °C) for 48 h; then, fermentation was carried out at room temperature (25 °C) or cooling temperature (4 °C) for 4 weeks. After, fermentation samples were dried at 45 °C for 48 h to a moisture content of approx. 10–12%. For analysis, the samples were ground in a mill (MK-06M, MPM, Milanówek, Poland). All samples were made in duplicate; the list of samples with markings is presented in Table 1. A scheme showing the layout of the pollen fermentation experiment is shown in Figure 1.

### 2.4. Microscopic Analysis

Microscopic analysis of the samples was performed in order to compare the morphology of pollen grains and to show changes occurring during the fermentation process. The observations were performed using an epifluorescence microscope BX-51 (Olympus, Shinjuku, Japan) equipped with a DP-72 digital camera and Cell D software. A water drop smear of each homogenized sample was made on a glass slide and observed at 200× magnification. 

### 2.5. Chemical Composition Analysis

#### 2.5.1. Physicochemical Parameters

Water activity for ground samples was measured using an HC2-AW probe and HW5 software (Rotronic AG, Bassersdorf, Switzerland) at a temperature 25 ± 2 °C.

The pH value and free acidity of pollen, bee bread, and fermented pollen samples were determined according to Shirsat et al. [17] using 10% *w*/*v* suspensions in deionized water. The pH value was measured with SevenCompact™ S210 pH-meter (Mettler Toledo, Columbus, OH, USA), while total free acidity was determined by titration with 0.1 M NaOH to reach a pH of 8.3. Acidity was converted to lactic acid according to Shirsat et al. [17] using the Formula (1):

(1) $$\text{Lactic acid content}=\left(\frac{V_{NaOH}\times C_{NaOH}\times 0.09}{m}\right)\times 100\%,$$

where: V_NaOH_—the volume of NaOH used to titration; C_NaOH_—NaOH titer; m—the mass of the sample; and 0.09—an equivalent weight of lactic acid.

#### 2.5.2. Inductively Coupled Plasma Optical Emission Spectroscopy (ICP-OES)

The content of minerals, including macroelements (Ca, K, Mg, Na, P, S), microelements (Al, Cr, Cu, Fe, Mn, Mo, Ni, Sr, Zn) and toxic elements (As, Cd, Pb), was determined in samples of pollen, bee bread, and fermented pollen using the ICP-OES method, according to the methodology described for propolis by Miłek et al. [18].

#### 2.5.3. Raman Spectroscopy

Ground samples of bee pollen, natural bee bread, and fermented bee pollen were used to analyze their chemical composition using the FT-Raman Nicolet NXR 9650 spectrometer (Thermo Fisher Scientific, Waltham, MA, USA) equipped with a 1064 nm laser. The samples were placed on the spectrometer table for the laser beam to fall on its center during the measurement. Measurements were performed at an aperture of 50 and a spectral resolution of 8 cm^−1^. The spectra were recorded in the range of 300 to 3500 cm^−1^ with a laser power of 0.5 W. For each spectrum, 64 scans were collected. Measurements were made in 5 replicates. Raman spectra were processed by the Omnic 8.1.11 (Thermo Fisher Scientific, Waltham, MA, USA) and OriginLab 2020 (Northampton, MA, USA) software.

#### 2.5.4. SDS-PAGE Electrophoresis

Protein profiles of selected samples were obtained by SDS-PAGE electrophoresis. Samples of pollen, bee bread, and fermented pollen were ground using an agate mortar. A total of 0.1 g of each sample was weighed and 300 µL of sample buffer was added to it, followed by sonication for 15 min. Then, centrifugation was performed at 14,500 rpm (MPW-55, MPW MED Instruments, Warsaw, Poland) and the supernatant was collected. Equal volumes of all protein extracts (15 µL) were applied to the gel wells. The separation was carried out according to the methodology described by Dżugan et al. [19]. A protein mass marker, ROTI^®^Mark BI-PINK (Carl Roth GmbH, Karlsruhe, Germany), was used to determine the molecular masses of proteins. 

### 2.6. In Vitro Biological Activity Tests

#### 2.6.1. Total Phenolic Content, Total Carotenoid Content, and Antioxidant Capacity

Extracts for the determination of the content of phenolic compounds and antioxidant capacity were prepared according to Kaškoniene et al. [12]. Two grams of each ground sample was suspended in 20 mL of 80% methanol and shaken for 24 h on an orbital shaker (Orbi-Shaker MP, Benchmark, Tempe, AZ, USA). The extract was filtered through filter paper. The residue was flooded again with 80% methanol and shaken again for 24 h. The obtained extracts were combined. 

Determination of the total content of phenolic compounds was performed by the Folin–Ciocalteu method adapted for measurement on a microplate reader, as described by Miłek et al. [18].

The total content of carotenoids was determined according to methods described by Kostić et al. [20] and based on extraction with 80% acetone and absorbance measurement at 450 nm. The total carotenoid content was calculated according to Equation (2):

Total carotenoid content (μg/g DW) = (A × V × 10^6^)/(E × 100 × m), (2)

where: A—absorbance of supernatant; V—total volume of the extract; E—specific extinction coefficient (2500); and m—mass of the sample. 

Antioxidant capacity was determined by FRAP and DPPH methods adapted for measurement in a microplate reader, as described earlier by Miłek et al. [18].

#### 2.6.2. Yeast Growth Assay

A liquid YPD medium was used for the growth of yeast cells (1% Difco Yeast Extract, 1% Yeast Bacto-Peptone, 2% (*w*/*v*) glucose) using a rotary shaker at 150 rpm, or a solid YPD medium containing 2% agar. The experiments were conducted at a temperature of 28 °C without (control) or with treatment of the yeast cells with tested samples. Data represent the average of three independent experiments.

The growth assay was conducted on a liquid YPD medium. The yeast cell suspensions were incubated at 28 °C for 24 h at 1200 rpm in a Heidolph Inkubator 1000 (Heidolph Instruments GmbH and Co, Schwabach, Germany). Growth was monitored turbidimetrically in the Anthos 2010 type 17,550 microplate reader at λ = 600 nm by performing measurements at 2 h intervals for 24 h. The data represent the mean values of three independent experiments.

#### 2.6.3. Spot Test 

The growth of yeast cultures was carried out in YPD medium until the exponential phase (OD600 nm between 0.4 and 0.5) and serially diluted according to the indicated concentrations (dilution ratio: 1:10, 1:100, 1:1000, 1:10,000). Five microliters of each cell suspension were spotted on YPD plates of agar. The growth of the cells was measured 48 h after incubation at 28 °C. At least three independent experiments were conducted to confirm each phenotype described in this paper. The spot plate assay was performed using the BY4741 strain and *sod1∆* treated with bee bread or pollen (preincubated for 2 h). The cells were then washed twice in sterile PBS and treated with 1 mM hydrogen peroxide for 1 h.

### 2.7. Statistical Analysis

Sample analyses were performed in triplicate, and results were presented as mean ± standard deviation. The results were subjected to one-way ANOVA and the significance of differences was determined based on Tukey’s test (*p* = 0.05) to determine the significant differences between bee pollen and fermented pollen as well as fermented pollen and natural bee bread. All quantified parameters were used as dependent variables and the type of sample was the independent variable in each case. All calculations were made using the Statistica 13.3 software (StatSoft, Tulsa, OK, USA). The principal components analysis (PCA) was used to compare samples for similarities and differences in their chemical composition detected by Raman spectroscopy. PCA was performed based on the whole FT-Raman spectra range of 300–3000 cm^−1^, which gave more than 900 variables. The reduction was conducted in such a way that the data with high variance were saved. PCA was processed by the OriginLab software (OriginLab Corporation, Northampton, MA, USA). 

## 3. Results and Discussion

### 3.1. Microscopic Analysis

**Figure 2:**
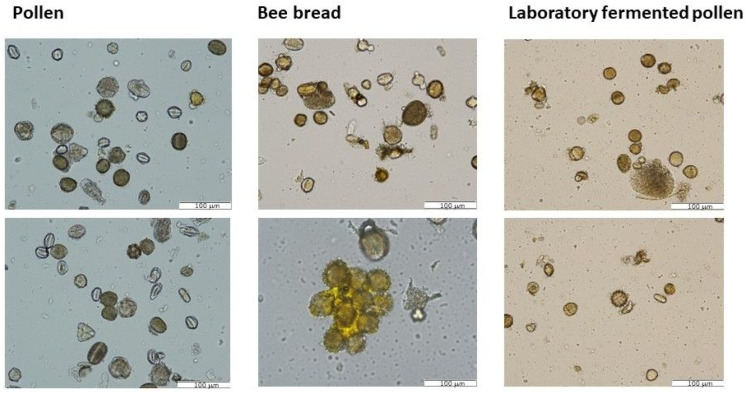
Comparison of microscopic images of natural bee pollen, natural bee bread (BB3), and laboratory-fermented bee pollen (FP25 U). The figure presents representative photographs (200× magnification).

Selected samples were subjected to microscopic observation in order to examine the effect of natural and induced fermentation on the morphology of plant pollen grains present in bee pollen (Figure 2). In the bee pollen sample, the presence of pollen grains typical for plants of the region of Central and Eastern Europe was found, namely, rapeseed (*Brassica napus*), goldenrod (*Solidago* sp.), black locust (*Robinia pseudoacacia*), or buckwheat (*Fagopyrum esculentum*). Pollen grains were well preserved despite the grinding of pollen loads; they are also not collected in larger clusters. In the case of natural bee bread (BB3), clusters of pollen grains were visible, and some of them show swelling. In the case of pollen fermented in laboratory conditions, both strongly fragmented grains and swollen grains showing intine spillage are visible. Similar observations regarding the presence of broken pollen grains and the release of internal content were made by Mora-Adames et al. [15], who also studied pollen fermentation on a laboratory scale. The use of ultrasound intensified grain destruction processes in the case of spontaneously fermented pollen (C25 U). 

### 3.2. Physicochemical Parameters

Easy-to-measure physicochemical parameters can help in the evaluation of the obtained products in comparison to both the original bee pollen and natural bee bread. The values of water activity, pH, and acidity (expressed as a percentage of lactic acid) are listed in Table 2.

Water activity is a parameter that determines the share of free water in the sample and allows for the assessment of the potential microbiological, chemical, and enzymatic stability of the product. For bee pollen, the water activity should not exceed 0.38 [21]; the starting pollen sample meets this requirement. For samples of fermented pollen, the value of this parameter is increased; it is significantly higher than in the tested samples of natural bee bread. This means that the samples obtained are more susceptible to the potential growth of xerotolerant microorganisms (e.g., some fungi, such as *Zygosaccharomyces*, *Metschnikowia* sp., and bacteria, such as *Bacillus* sp.) that can exist at low water activity values [22] and the occurrence of other unfavorable changes. 

As a result of the fermentation process, a significant decrease in pH and an increase in acidity were observed in relation to the starting pollen sample. The pH value reached approximately half the distance from the average value for natural bee bread (4.08). Changes in both parameters confirm partial fermentation of bee pollen. However, the positive effect of ultrasound treatment and introducing the starter culture was not observed. It is well known that bee pollen has a higher pH value than bee bread, but it is quite diverse depending on geographical and botanical origin. Bee pollen samples from various regions of Lithuania and other European countries examined by Adaškevičiūte et al. showed a pH range of 4.30 to 5.22. Bee bread tested by the same authors showed pH values between 4.11 and 4.37 [13]. Similarly, pollen from different plants obtained from different regions of Brazil ranged from 4.55 to 5.09 [23]. The dependence of the pH decrease on the type of starter culture used for pollen fermentation was observed by Poyraz et al. [24], and the largest decrease (from 5.13 to 4.15) was noted for the sample fermented with the fructophilic lactic acid bacteria (FLAB), *Lactobacillus kunkeei*, and the yeasts *Starmeralla magnolia* (MP-2) and *Zygosaccharomyces siamensis* (MP-14). The pH value can be considered a simple indicator of the course of pollen fermentation, indicating the presence of organic acids in its process. Acidity expressed as lactic acid percentage increased by almost 50% compared to the control. The final score is lower than that of Shirsat et al. [17], who, in the pollen fermentation process, recorded an increase from 4.12 to 6.1% after 168 h of the process. However, they used pollen with a higher initial content of lactic acid (4.12%) and the overall increase is similar to the described experiment. In the case of pollen fermentation with the participation of various microorganisms, it was possible to achieve a varied content of lactic acid: from 0.45 to 4.40 mg/g [24]. 

### 3.3. Chemical Composition

#### 3.3.1. Mineral Content

The comparison of the mineral composition of the tested initial pollen, fermented products, and selected natural bee bread samples is shown in Table 3. Comparing the results of fermented pollen to the initial substrate, a significant decrease in the content of the main macroelements, Ca, K, Na, P, Mg, and S, can be observed (*p* < 0.05). At the same time, there was an increase in the content of some microelements, especially Cu, Ni, Cr, and Fe. The observed changes may be the result of adding honey, water, and bacteria to the matrix, but confirmation of such ideas requires further studies with appropriate controls. Moreover, comparing the mineral composition of products fermented under different conditions (temperature, ultrasound pre-treatment, LAB inoculation) it is difficult to determine a clear trend, and the observed few significant changes are rather accidental and cannot be related to the course of the process. Similarly, an increase in the content of copper and iron, and additionally also magnesium, manganese, and zinc as a result of pollen fermentation, was observed by Shirsat et al. [17]. The presence of toxic elements, Cd, Pb, and As, was not recorded in any of tested samples, which confirms the high quality of tested samples free of environmental contaminants.

In turn, the mineral composition of natural bee bread was more abundant than the obtained fermented substitute, as well as raw pollen. In general, bee bread contained more calcium, potassium, magnesium, and phosphorus on average. The higher content of the main elements, Mg and Ca, in bee bread is confirmed in the results of other studies [25]. Among microelements, significant differences were observed for chromium—present in pollen-based samples in the greater amount—of 0.16 to 0.41 mg/100 g. However, a direct comparison is not fully reliable due to possible differences in the mineral composition of pollen processed by bees and transformed in laboratory conditions. Literature data confirm that the mineral composition of both bee pollen and bee bread is quite diverse [25,26]. The mineral composition of pollen, as well as bee pollen, may be strongly influenced not only by botanical origin but also by geographical conditions, especially soil as well as apicultural practices and environmental pollution [27,28]. The richness of basic elements (Ca, K, P, Na, Mg, Mn, Zn) is important for bees, for which bee pollen is the main source of these essential elements [28].The content of individual elements in bee bread is highly differentiated depending on the geographical origin, which is also related to the composition of the flora used by bees [5]. However, the role of the bee family in the conversion of pollen to bee bread cannot be overlooked, as evidenced by the great diversity of three local samples tested coming from the same region with similar flora. 

#### 3.3.2. Raman Spectroscopy

**Figure 3:**
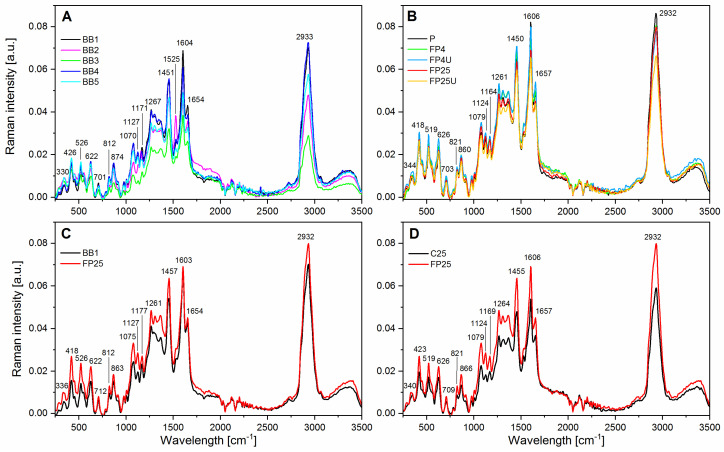
Raman spectra of the bee pollen and natural bee bread with the regions corresponding to vibrations of functional groups. (**A**)—natural bee bread from five different locations (BB1-5); (**B**)—comparison of initial natural bee pollen (P) and laboratory-fermented pollen (FP4, FP4 U, FP25, and FP25 U); (**C**)—comparison of the best laboratory-fermented pollen and natural bee bread; (**D**)—comparison of spontaneously (C25) and inoculated (FP25) laboratory-fermented pollen.

In all analyzed samples of bee pollen and natural bee bread, the presence of bands characteristic of fructose, glucose, phenolic compounds, flavonoids, carotenoids, proteins, and fatty acids was found (Figure 3). Band ranges in the Raman spectra corresponding to individual groups of compounds identified in the tested samples are shown in Table 4.

**Figure 4:**
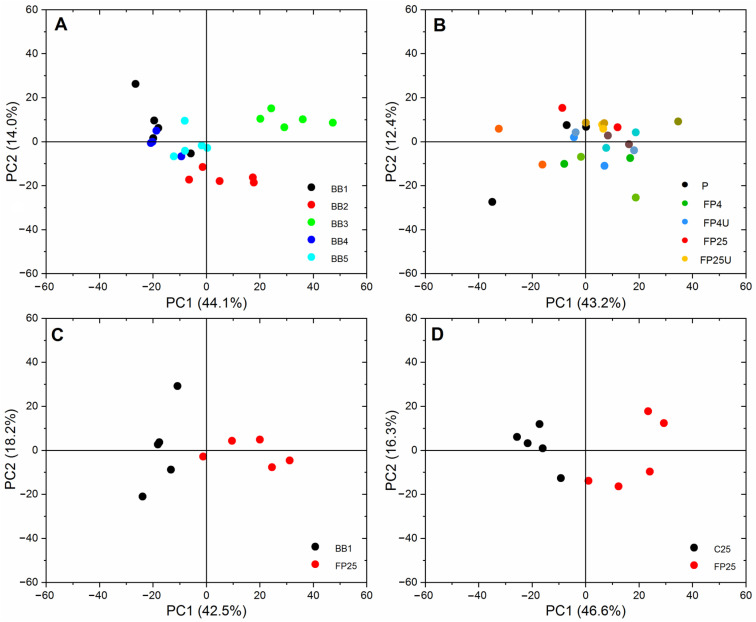
The 2D score graphs for PCA of FT-Raman data (300–3000 cm^−1^) of bee pollen and natural bee bread. (**A**)—natural bee bread from five different locations (BB1–5); (**B**)—comparison of initial natural bee pollen (P) and laboratory-fermented pollen (FP4, FP4 U, FP25, and FP25 U); (**C**)—comparison of the best laboratory-fermented pollen and natural bee bread; (**D**)—comparison of spontaneously (C25) and inoculated (FP25) laboratory-fermented pollen.

Differences between subjects were solely due to lower or higher content of a given chemical substance in a particular sample. The greatest changes were noticed in the case of the BB2 and BB3 bee bread (Figure 4A). A relatively unique sample turned out to be BB2, which stood out from the others due to the presence of an additional band from carotenoids at 1520 cm^−1^ (Figure 3A). The unique carotenoid composition of BB2 most likely results from its location and specific vegetation. It was previously reported that the geographical origin of natural bee bread had a significant impact on its chemical composition [5]. A comparison of natural pollen with pollen fermented in laboratory conditions showed that combined higher temperature and ultrasound (FP25 U) reduced the content of lipids (2932 cm^−1^), phenols, and flavonoids (bands in the range of 1264–1365 cm^−1^) (Figure 3B). However, using low-temperature fermentation and ultrasound treatment (FP4 U) increased the content of phenols and flavonoids (Figure 3B and Figure 4B). Pollen fermented at a higher temperature (25 °C) was characterized by a higher content of lipids, sugars, phenols, and flavonoids, to compare natural bee bread (Figure 3C) and pollen obtained in controlled inoculation with bacteria (Figure 3D and Figure 4D). The scatter plot of PC1 (variability between 42% and 47%) and PC2 (variability between 12% and 18%) shows that the laboratory-fermented pollen from 25 C (FP25) and natural bee bread from three locations (BB3) were significantly different from other objects (Figure 4A–D).

#### 3.3.3. SDS-PAGE Electrophoresis

**Figure 5:**
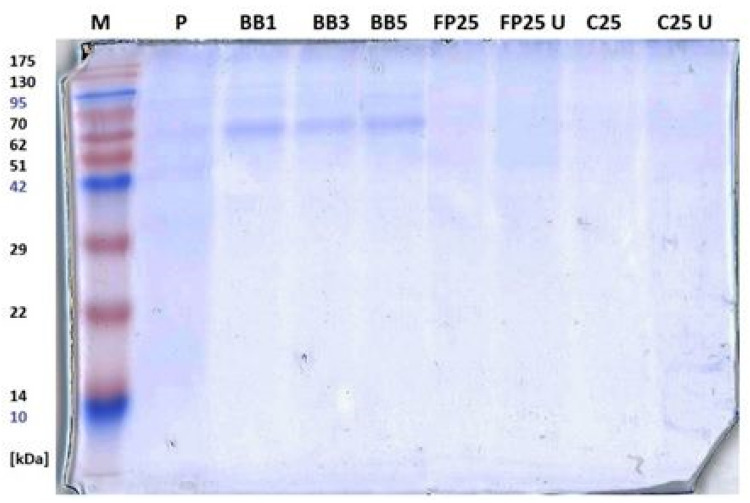
SDS PAGE gel image. M—molecular mass marker; P—bee pollen; BB1, BB3, BB5—natural bee bread; FP25, FP25 U, C25, C25 U—laboratory-fermented bee pollen.

Protein profiles obtained for selected samples by SDS PAGE are shown in Figure 5.

The protein profile obtained for the original pollen differs from that of natural bee bread. Bands of proteins with masses of approx. 75, 65, and 45 kDa are poorly visible, while in all tested samples of natural bee bread, the band corresponding to the protein with a mass of approx. 65 kDa clearly dominates. It may be identified as a bee-derived protein introduced by honeybees when processing pollen into bee bread. The mass (65 kDa) would correspond to bee alpha-glucosidase, which was previously identified in honey [29]. This would explain the lack of this intense bands in samples that were formed without the participation of bees. The samples of laboratory-fermented pollen do not show clear differences in relation to the initial bee pollen, at least in the concentration used in the electrophoretic analysis. The higher intensity of the bands in the paths corresponding to the natural bee bread suggests a higher protein content in these samples. Literature data report similar protein profiles for both bee pollen and bee bread with a predominant fraction of proteins between 20 and 85 kDa [30,31]. These are most likely proteins of plant origin, typical of pollens of various plant species. Another study has shown that regardless of the source of pollen, the protein composition of natural bee bread is conservative [31].

Based on the parameters of the chemical composition of the laboratory-converted pollen, it can be concluded that regardless of the composition of the fermentation microflora (*L. rhamnosus* and/or native bee pollen microorganisms, also including certain species of yeast), the process incompletely reproduces the conditions of bee bread production in the hive. It seems that the complete conversion of pollen into bee bread is not only determined by the fermentation process, but also by other biochemical transformations catalyzed by bee-derived enzymes. Kaškoniene et al. [12] also did not observe significant differences between samples of Lithuanian pollen fermented with *L. rhamnosus* and without additional inoculation. They explained it by the decisive role of native pollen microflora in the conversion process. Moreover, the native microorganisms that occurred in bee pollen were not destroyed during applied sonication.

### 3.4. In Vitro Biological Activity Tests

#### 3.4.1. Antioxidant Activity

The relationship between the total content of phenolic substances, carotenoids, and antioxidant activity measured by FRAP and DPPH methods are presented in Table 5. The polyphenolic content was comparable in all samples studied (*p* > 0.05), excluding spontaneously fermented pollen at 25 °C. Despite the carotenoid content and reducing power, FRAP was positively affected by the fermentation process in higher temperature in both cases (spontaneously and controlled). However, the unequivocal impact of ultrasound pre-treatment and LAB inoculation cannot be defined. 

Bee pollen used as a substrate for fermentation contained 10.36 mg/g of phenolic compounds and 24.96 µg of carotenoids. The average content of total polyphenols in pollen varies depending on the botanical origin. Rajs et al. [32], in monovarietal pollen studies, obtained values between 4 and 15.80 mg GAE/g. Other studies indicate a higher content of this fraction of bioactive compounds, i.e., 32.52 or 27.03 mg GAE/g for bee pollen from Poland [33,34]. The content of carotenoids is also variable, which is visible in the colors of individual grains of pollen loads, depending on the plant from which the pollen is obtained by bees. The high content of carotenoids in the BB2 sample was observed above in the Raman spectrum. Literature data report carotenoid content between 27.08 to 344.6 µg/g for fresh pollen and from 25.34 to 268.5 µg/g for processed [35]. For the tested samples of bee bread, the content of phenolic compounds was significantly lower than in pollen, i.e., 9.53 mg GAE/g, and carotenoid content was higher, i.e., 33.14 µg/g on average. Samples of Polish bee bread studied by Sawicki et al. contained an average of 8.23 mg GAE/g of phenolic compounds. The range of phenolic content in bee bread is also very wide and can range from 0.7 to 136 mg GAE/g [33]. A lower average content of phenolic compounds, as well as antiradical activity, was observed in natural bee bread than in bee pollen. This observation is confirmed by other authors who compared bee bread and pollen from the same areas [28,33]. The opposite trend was observed in the case of the reducing power of FRAP, which is also confirmed by other studies [33]. The comparison of pollen fermented in laboratory conditions to natural bee bread favors the artificial bee bread. It may be related to different pollen used for fermentation. Other authors have observed an increase in the content of phenolic compounds in pollen as a result of fermentation, both spontaneous and induced by lactic acid bacteria, achieving higher results for the content of flavonoids than for natural bee bread. As in our case, a decrease in the ability to scavenge the DPPH radical was also observed by fermented pollen samples [12]. A decrease in antioxidant activity as a result of the fermentation process was also observed for other products, e.g., cocoa beans [36]. The antioxidant activity of bee pollen is extremely diverse depending on the botanical origin. Different plant pollens contain different phytochemicals responsible for their antioxidant potential. The diversity is also the result of the use of different extraction systems and methods of analysis [37]. The same is true for bee bread, whose antioxidant properties are also varied [5,28]. Among the pollen fractions of individual plants isolated from bee pollen, pollens of plants such as *Sinapis alba*, *Phacelia tanacetifolia*, *Robinia pseudoacacia*, and *Aesculus hippocastanum* were distinguished by a particularly high antioxidant activity, while pollens of *Zea mays*, *Lamium purpureum*, and *Chamerion angustifolium* showed much weaker properties [38]. Changes in the antioxidant properties of pollen as a result of fermentation can be explained by the release of antioxidant substances as a result of the destruction of the structure of pollen grains, which could also be due to the use of pollen sonication.

#### 3.4.2. Yeast Growth Assay

**Figure 6:**
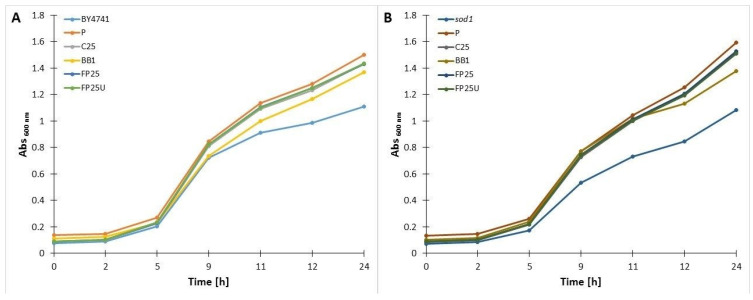
Growth kinetics of the wild-type yeast BY4741 (**A**) and the isogenic mutant *sod1∆* (**B**) treated with bee bread or pollen The optical density (OD600 nm) of the culture was measured for 24 h at different points. The data presented are replicates from two independent cultures ± SD, which are smaller or equal in size to the symbol size.

Several physiological and molecular mechanisms have been identified by studying the growth of the budding yeast *Saccharomyces cerevisiae*. Two methods are primarily used to measure growth: serial dilutions for spot testing and growth curves. To investigate specific responses or phenotypes, these techniques can be combined with a wide variety of substrates, environmental conditions, mutants, and chemicals. The growth rate of yeast cells treated with bee pollen is shown in Figure 6. In this study, the reference strain, BY4741, as well as a mutant lacking superoxide dismutase, the *sod1∆* deletion mutant, were used. As expected, an analysis of the growth rate of untreated *sod1∆* shows a slowdown in the doubling time compared to the wild type. As an interesting note, we have shown in this study that yeast cells exposed to tested samples, both wild-type and *sod1∆*, have a significantly accelerated growth rate compared to cells untreated with bee pollen. Treatment with P1 was associated with a particularly high doubling rate. It is noteworthy that the analyzed strains revealed an increase in maximum optical density, which is indicative of changes in cellular metabolism. Furthermore, for the first 12 h of the experiment, we did not observe significant differences in growth rate between the wild strain and *sod1∆* treated with pollen or bee bread. Therefore, the addition of bee bread or pollen abolishes the phenotypic effect of the lack of superoxide dismutase, which is a key observation in the context of the use of bee bread as an antioxidant preparation. Although the mechanism by which this interesting phenotype is formed is not fully understood, we have hypothesized that it might be caused by certain metabolites present in bee bread, such as lactic acid.

**Figure 7:**
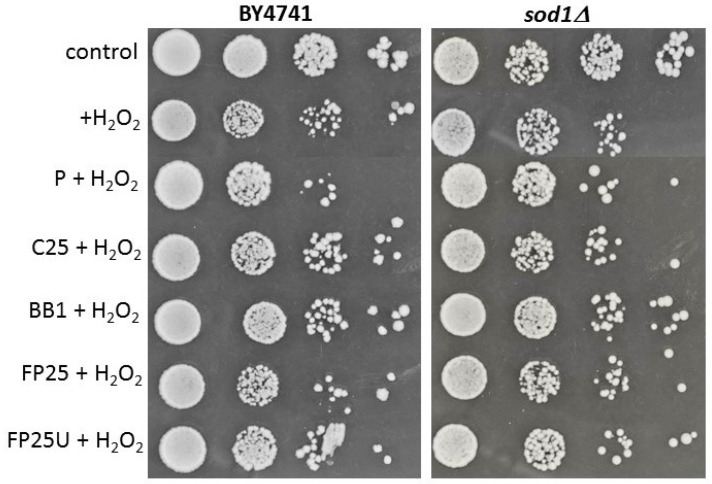
Spot plate assay of the BY4741 (WT) and isogenic mutant *sod1Δ* preincubate with bee bread or pollen (2 h) and additionally treated with 1 mM hydrogen peroxide for 2 h. Strains were serially diluted (10^6^ to 10^3^). Control indicates growth on rich YPD medium. Each spot assay was performed at least in triplicate.

Then, it was asked whether the pollen or bee bread used has a protective ability against hydrogen peroxide, the main precursor of oxygen free radicals in yeast cells. As shown in Figure 7, in the case of the *sod1Δ* mutant, the key contribution of P, BB1, and FP25 U in protection against free radicals was observed. 

It has been demonstrated in our previous research that yeast can also be used to study other natural substances. Recent studies have investigated the antioxidant properties of honeys enriched with *A. melanocarpa* L. in vivo using a yeast model. Using modified honey as a pretreatment agent protects yeast cells from oxidative stress induced by hydrogen peroxide [39]. Over the last few years, yeast has been used as an interesting eukaryotic model to study the effects of many substances on the metabolism of cells, including coffee [40], Kombucha [41], and many others.

## 4. Conclusions

The applied experimental conditions resulted in the partial fermentation of bee pollen. The obtained product showed intermediate properties between the initial pollen and bee bread, i.e., pH value and lactic acid content. However, higher content of polyphenols and comparable antioxidant activity to natural bee bread were observed. A more bee-bread-like product regarding acidity was produced at a higher fermentation temperature (25 °C), while the effect of ultrasound treatment and starter culture inoculation seems to have had no positive effect. The yeast model’s stronger capability to promote yeast growth rate and its protective effect against hydrogen peroxide-induced oxidative stress conditions have been shown. The laboratory process of bee pollen fermentation requires further optimization, but it seems to be a promising imitation of the formation of bee bread in the hive.

## Figures and Tables

**Table 1:** Tested variants of pollen fermentation.

Sample Symbol	Temperature of Incubation	Ultrasound Treatment	Bacteria Inoculation
FP4	4 °C	-	3 × 10^9^ CFU
FP4 U	4 °C	2 × 15 min	3 × 10^9^ CFU
FP25	25 °C	-	3 × 10^9^ CFU
FP25 U	25 °C	2 × 15 min	3 × 10^9^ CFU
C4	4 °C	-	-
C4 U	4 °C	2 × 15 min	-
C25	25 °C	-	-
C25 U	25 °C	2 × 15 min	-

FP—fermented pollen; C—control (not inoculated); U—ultrasound treatment.

**Table 2:** Water activity, pH, and lactic acid content of analyzed samples.

Sample	Water Activity	pH	Lactic Acid Content [%]
Bee pollen			
P	0.3539 ± 0.0019 ^a^	5.01 ± 0.06 ^a^	2.49 ± 0.31 ^a^
Fermented pollen			
FP4	0.4663 ± 0.0010 ^f^	4.58 ± 0.05 ^de^	3.23 ± 0.20 ^c^
FP4 U	0.4866 ± 0.0014 ^g^	4.63 ± 0.02 ^df^	2.78 ± 0.12 ^ae^
C4	0.4667 ± 0.0005 ^f^	4.67 ± 0.01 ^f^	3.14 ± 0.18 ^ace^
C4 U	0.4475 ± 0.0005 ^b^	4.68 ± 0.01 ^f^	3.51 ± 0.06 ^c^
FP25	0.4650 ± 0.0009 ^f^	4.55 ± 0.04 ^eg^	3.50 ± 0.32 ^c^
FP25 U	0.5343 ± 0.0020 ^h^	4.50 ± 0.05 ^g^	3.22 ± 0.31 ^ce^
C25	0.5388 ± 0.0014 ^i^	4.52 ± 0.00 ^g^	3.25 ± 0.09 ^ce^
C25 U	0.4768 ± 0.0003 ^j^	4.52 ± 0.02 ^g^	3.69 ± 0.06 ^c^
Natural bee bread			
BB1	0.4451 ± 0.0019 ^b^	3.99 ± 0.01 ^b^	4.70 ± 0.43 ^b^
BB2	0.4373 ± 0.0012 ^c^	3.99 ± 0.04 ^b^	4.95 ± 0.30 ^b^
BB3	0.4992 ± 0.0003 ^d^	4.14 ± 0.01 ^c^	3.24 ± 0.46 ^c^
BB4	0.4112 ± 0.0005 ^e^	4.17 ± 0.00 ^c^	3.82 ± 0.38 ^cd^
BB5	0.4136 ± 0.0009 ^e^	4.10 ± 0.03 ^c^	4.47 ± 0.01 ^bd^
Natural bee bread (average)	0.4412 ± 0.0329	4.08 ± 0.08	4.24 ± 0.70

^a,b,c,d,e,f,g,h,i,j^ means values sharing the same letters (in a column) are not significantly different (*p* > 0.05). P—bee pollen; FP4–C25 U—laboratory-fermented pollen (symbols as given in Table 1); BB1–BB5—natural bee bread.

**Table 3:** Mineral composition of analyzed samples.

Mineral Content [mg/100 g]	P	FP4	C4	FP25	C25	BB1	BB3	BB5
Macroelements								
P	631.52 ± 8.70 ^a^	498.70 ± 3.91 ^b^	455.65 ± 3.71 ^c^	594.86 ± 4.96 ^d^	527.10 ± 5.89 ^e^	855.14 ± 5.87 ^f^	836.16 ± 2.80 ^g^	956.30 ± 7.81 ^h^
K	465.80 ± 3.53 ^a^	427.68 ± 1.20 ^b^	420.80 ± 2.75 ^b^	411.52 ± 2.88 ^c^	421.59 ± 2.44 ^b^	548.12 ± 4.47 ^d^	508.12 ± 3.65 ^e^	561.88 ± 0.33 ^f^
S	218.55 ± 0.33 ^a^	191.21 ± 0.39 ^b^	184.28 ± 0.71 ^c^	194.70 ± 0.83 ^d^	192.07 ± 1.00 ^b^	195.41 ± 0.44 ^d^	203.99 ± 1.39 ^e^	251.96 ± 0.43 ^f^
Ca	166.43 ± 2.15 ^a^	143.23 ± 3.27 ^bc^	135.62 ± 1.89 ^b^	150.11 ± 2.10 ^c^	145.59 ± 0.39 ^bc^	205.41 ± 3.49 ^d^	207.31 ± 6.25 ^d^	226.96 ± 691 ^e^
Na	3.37 ± 0.10 ^a^	2.01 ± 0.19 ^b^	1.43 ± 0.17 ^c^	4.15 ± 0.30 ^d^	2.56 ± 0.16 ^e^	4.68 ± 0.17 ^f^	9.30 ± 0.23 ^g^	11.79 ± 0.10 ^h^
Mg	91.78 ± 0.87 ^a^	87.38 ± 0.20 ^b^	88.59 ± 0.61 ^b^	81.59 ± 0.68 ^c^	83.91 ± 0.62 ^c^	173.58 ± 4.47 ^d^	114.47 ± 1.12 ^e^	101.10 ± 0.05 ^f^
Microelements								
Fe	5.82 ± 1.15 ^a^	9.22 ± 0.58 ^b^	5.96 ± 0.70 ^a^	23.72 ± 0.11 ^c^	12.62 ± 0.84 ^b^	2.49 ± 0.38 ^d^	5.32 ± 0.30 ^a^	10.99 ± 0.51 ^b^
Zn	4.32 ± 0.00 ^a^	3.26 ± 0.0 ^b^	3.07 ± 0.01 ^c^	3.16 ± 0.00 ^d^	3.31 ± 0.01 ^e^	2.25 ± 0.01 ^f^	2.72 ± 0.02 ^g^	3.22 ± 0.01 ^h^
Al	3.56 ± 0.43 ^ab^	3.96 ± 0.13 ^ab^	3.21 ± 0.46 ^b^	3.43 ± 0.23 ^ab^	3.30 ± 0.11 ^ab^	1.47 ± 0.30 ^c^	3.03 ± 0.38 ^a^	7.06 ± 0.32 ^d^
Mn	2.59 ± 0.02 ^a^	2.32 ± 0.08 ^bc^	2.36 ± 0.06 ^b^	2.16 ± 0.02 ^c^	2.30 ± 0.04 ^bc^	2.25 ± 0.08 ^bc^	2.55 ± 0.04 ^a^	3.01 ± 0.06 ^d^
Ni	0.23 ± 0.01 ^ab^	0.34 ± 0.01 ^c^	0.28 ± 0.00 ^cd^	1.22 ± 0.01 ^e^	0.50 ± 0.02 ^f^	0.20 ± 0.01 ^a^	0.24 ± 0.02 ^bd^	0.26 ± 0.01 ^bd^
Cr	0.16 ± 0.10 ^ab^	0.37 ± 0.16 ^b^	0.41 ± 0.06 ^b^	0.33 ± 0.08 ^b^	0.37 ± 0.04 ^b^	0.07 ± 0.13 ^a^	0.02 ± 0.04 ^a^	0.02 ± 0.03 ^a^
Mo	0.16 ± 0.01 ^a^	0.16 ± 0.01 ^a^	0.17 ± 0.01 ^a^	0.17 ± 0.01 ^a^	0.16 ± 0.01 ^a^	0.16 ± 0.00 ^a^	0.18 ± 0.01 ^a^	0.18 ± 0.00 ^a^
Cu	0.12 ± 0.02 ^a^	0.47 ± 0.01 ^b^	0.21 ± 0.02 ^c^	2.41 ± 0.01 ^d^	1.69 ± 0.03 ^e^	0.09 ± 0.01 ^a^	0.21 ± 0.03 ^c^	0.26 ± 0.02 ^c^
Sr	0.15 ± 0.00 ^a^	0.14 ± 0.00 ^b^	0.13 ± 0.00 ^b^	0.13 ± 0.00 ^b^	0.13 ± 0.01 ^b^	0.12 ± 0.00 ^c^	0.17 ± 0.00 ^d^	0.19 ± 0.00 ^e^
Toxic elements								
As	0.00 ± 0.01 ^a^	0.00 ± 0.00 ^a^	0.01 ± 0.02 ^a^	0.00 ± 0.01 ^a^	0.00 ± 0.00 ^a^	0.00 ± 0.00 ^a^	0.00 ± 0.00 ^a^	0.00 ± 0.01 ^a^
Cd	0.00 ± 0.00 ^a^	0.00 ± 0.00 ^a^	0.00 ± 0.00 ^a^	0.01 ± 0.00 ^a^	0.01 ± 0.00 ^a^	0.01 ± 0.00 ^a^	0.01 ± 0.00 ^a^	0.01 ± 0.00 ^a^
Pb	0.00 ± 0.00 ^a^	0.03 ± 0.02 ^a^	0.00 ± 0.00 ^a^	0.00 ± 0.00 ^a^	0.00 ± 0.00 ^a^	0.01 ± 0.01 ^a^	0.01 ± 0.01 ^a^	0.00 ±0.00 ^a^

^a,b,c,d,e,f,g,h^—means sharing the same letters (in a row) are not significantly different (*p* > 0.05). P—bee pollen; FP4–C25 U—laboratory-fermented pollen (symbols as given in Table 1); BB1–BB5—natural bee bread. Data marked in green indicate a significant increase and in red a significant decrease in relation to bee pollen as a result of fermentation.

**Table 4:** A listing of the positions of the Raman bands identified in bee pollen and natural bee bread with the description of vibrations corresponding to the respective functional groups.

Characteristic Vibrations for the Functional Groups	Range of Peak Positions [Raman Shift, cm^−1^]
fructose	418–426; 519–526; 860–874;
glucose	1070–1079; 1124; 1127;
phenols	1261–1267;
flavonoids	1360; 1450–1457;
carotenoids	1164–1171; 1525;
lipids	1654–1657; 2933;
proteins	1603–1606;

**Table 5:** Total phenolic, carotenoid content, and antioxidant potential of tested samples.

Sample	Total Phenolic Content [mg GAE/g]	Total Carotenoid Content [μg/g]	Antioxidant Potential [μmol TE/g]	
			FRAP	DPPH
Bee pollen				
P	10.36 ± 0.48 ^ad^	24.96 ± 0.57 ^a^	26.61 ± 0.25 ^a^	17.61 ± 0.52 ^a^
Fermented pollen				
FP4	10.29 ± 0.17 ^ad^	25.72 ± 0.40 ^ag^	28.40 ± 0.28 ^ad^	14.36 ± 0.37 ^b^
FP4 U	10.43 ± 0.16 ^ad^	23.96 ± 0.06 ^b^	29.80 ± 1.01 ^ae^	15.44 ± 0.32 ^b^
C4	10.81 ± 0.05 ^a^	29.08 ± 0.17 ^c^	30.70 ± 0.48 ^b^	15.54 ± 0.21 ^b^
C4 U	11.07 ± 0.34 ^a^	24.88 ± 0.11 ^a^	30.06 ± 1.26 ^be^	16.22 ± 0.14 ^c^
FP25	10.36 ± 0.05 ^ad^	28.16 ± 0.11 ^c^	29.81 ± 0.79 ^a^	14.85 ± 0.12 ^b^
FP25 U	10.24 ± 0.19 ^ad^	27.44 ± 0.34 ^c^	29.96 ± 1.35 ^b^	14.99 ± 0.62 ^b^
C25	11.35 ± 0.10 ^b^	35.84 ± 0.11 ^d^	33.21 ± 0.89 ^b^	16.07 ± 0.38 ^c^
C25 U	10.99 ± 0.25 ^b^	28.08 ± 0.11 ^c^	32.38 ± 2.17 ^b^	15.36 ± 0.18 ^b^
Natural bee bread				
BB1	8.86 ± 0.48 ^c^	27.48 ± 0.17 ^c^	26.27 ± 1.85 ^cd^	15.94 ± 0.49 ^c^
BB2	9.36 ± 0.24 ^cd^	43.32 ± 1.30 ^e^	24.20 ± 1.03 ^c^	14.17 ± 0.14 ^b^
BB3	10.23 ± 0.45 ^a^	37.56 ± 0.17 ^f^	28.51 ± 0.81 ^de^	17.06 ± 0.80 ^a^
BB4	9.52 ± 0.21 ^d^	26.04 ± 0.17 ^g^	27.95 ± 0.83 ^cde^	15.42 ± 0.28 ^b^
BB5	9.68 ± 0.04 ^d^	31.28 ± 0.34 ^h^	27.97 ± 0.35 ^cde^	16.58 ± 0.65 ^ac^
Natural bee bread (average)	9.53 ± 0.50	33.14 ± 7.23	26.98 ± 1.77	15.83 ± 1.12

^a,b,c,d,e,f,g,h^–means sharing the same letters (in a column) are not significantly different (*p* > 0.05). P—bee pollen; FP4–C25 U;—laboratory-fermented pollen (symbols as given in Table 1); BB1–BB5—natural bee bread.

## Data Availability

The data presented in this study are available in the article.
